# Investigating Material Interface Diffusion Phenomena through Graph Neural Networks in Applied Materials

**DOI:** 10.1021/acsami.4c10240

**Published:** 2024-09-18

**Authors:** Zirui Zhao, Hai-Feng Li

**Affiliations:** Institute of Applied Physics and Materials Engineering, University of Macau, Avenida da Universidade, Taipa, Macao SAR 999078, China

**Keywords:** Graph neural networks (GNNS), interface diffusion, material properties prediction, atomic structure modeling, semiconductor interfaces

## Abstract

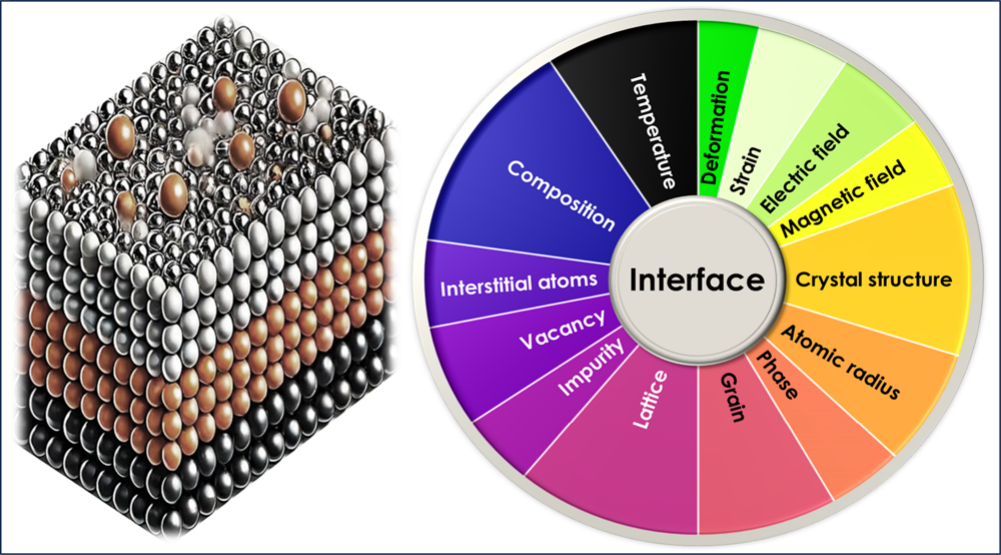

Understanding and predicting interface diffusion phenomena in materials is crucial for various industrial applications, including semiconductor manufacturing, battery technology, and catalysis. In this study, we propose a novel approach utilizing Graph Neural Networks (GNNs) to investigate and model material interface diffusion. We begin by collecting experimental and simulated data on diffusion coefficients, concentration gradients, and other relevant parameters from diverse material systems. The data are preprocessed, and key features influencing interface diffusion are extracted. Subsequently, we construct a GNN model tailored to the diffusion problem, with a graph representation capturing the atomic structure of materials. The model architecture includes multiple graph convolutional layers for feature aggregation and update, as well as optional graph attention layers to capture complex relationships between atoms. We train and validate the GNN model using the preprocessed data, achieving accurate predictions of diffusion coefficients, diffusion rates, concentration profiles, and potential diffusion pathways. Our approach offers insights into the underlying mechanisms of interface diffusion and provides a valuable tool for optimizing material design and engineering. Additionally, our method offers possible strategies to solve the longstanding problems related to materials interface diffusion.

## 1 Introduction

Materials science is a pivotal discipline driving modern technological advancements, focusing on the composition, structure, properties, and relationships of materials. Within materials engineering, interface diffusion profoundly influences mechanical properties, conductivity, and thermal stability, impacting various practical applications.^1,2^ However, traditional research methods, relying on experimental techniques and numerical simulations, often struggle with complex multiphase interface problems due to their time-consuming and labor-intensive nature.^3^ Despite significant advancements in materials science and engineering, many unresolved challenges persist in the study of interfacial diffusion. The complexity of interfacial phenomena, driven by diverse material properties and environmental conditions, continues to elude comprehensive understanding. Moreover, the heterogeneous nature of materials and the multiscale interactions involved further complicate accurate prediction and control of diffusion processes at interfaces.

To address these challenges, it is essential to identify specific unresolved issues related to material interface diffusion in various material systems, as detailed in Table 1. These issues are critical in the context of materials science and engineering, especially in applications involving semiconductors, electronic devices, solid-state batteries, and thin-film technologies.^4−7^ For instance, the diffusion at the interface between silicon (Si) and silicon dioxide (SiO_2_) significantly affects the electrical properties in semiconductor manufacturing, posing challenges in enhancing device performance.^8^ Similarly, the electromigration phenomenon due to copper (Cu) interconnect diffusion in silicon (Si) impacts circuit stability and resistance, requiring advanced strategies to mitigate these effects.^9^ In the case of germanium-doped silicon (Ge–Si), understanding the interface diffusion is essential for managing crystal defects and optimizing electronic properties.^10^ Additionally, multilayer nitride films on silicon need careful examination of diffusion impacts to maintain film stress and structural stability.^11^ High-power electronic devices benefit from controlled silicon and silicon carbide (SiC) interface diffusion,^12^ while the dielectric properties and adhesion of aluminum oxide (Al_2_O_3_) thin films depend on minimizing interface diffusion with silicon.^13^ Furthermore, the performance of high-frequency devices can be compromised by the diffusion at the silicon and gallium nitride (GaN) interface.^14^ The contact resistance and reliability issues arising from gold (Au) diffusion in silicon,^15^ along with the electrical properties of nickel silicide (NiSi) affected by nickel (Ni) diffusion, highlight the need for precise interface management.^11^ Finally, optimizing the performance of optoelectronic devices involves addressing silicon and indium phosphide (InP) interface diffusion challenges.^16^ These examples underscore the importance of advanced research and innovative solutions in managing material interface diffusion to enhance the performance and reliability of various technological applications.

**Table 1:** Summary of Unresolved Issues Related to Material Interface Diffusion (ID)^a^

Material interfaces	Unresolved issues	Refs.
SSEs and cathodes	Strategies to reduce interfacial resistance	(4)
Si and SiO_2_	Investigating the impact of SiO_2_–Si interface diffusion on electrical properties	(8)
Cu and Si	Examining the electromigration phenomenon due to Cu interconnect diffusion in Si	(9)
Ge and Si	Assessing how Ge–Si interface diffusion affects crystal defects	(10)
Si and ML Ni	Analyzing the impact of nitride diffusion on film stress and structural stability	(11)
Si and Ni	Optimizing the formation and electrical properties of nickel silicide (NiSi)	(11)
Si and SiC	Investigating Si–SiC interface diffusion to enhance performance in high-power devices	(12)
Si and Al_2_O_3_	Enhancing dielectric properties and adhesion of Al_2_O_3_ thin films	(13)
Si and GaN	Examining the impact of interface diffusion on high-frequency devices	(14)
Si and Au	Addressing increased contact resistance issues and reliability due to Au diffusion	(15)
Si and InP	Investigating the impact of Si–InP interface diffusion on optoelectronic devices	(16)
p- and n-type SCs	Exploring methods to minimize ID affecting electrical and thermal conductivities	(51)

a Note: SSEs = solid-state electrolytes, SCs = semiconductors, ML = multilayer, Refs. = References.

In recent years, the rapid advancement of artificial intelligence, particularly in the realm of machine learning, offers promising avenues for materials science. Graph Neural Networks (GNNs), adept at handling noneuclidean data such as graph-structured data, have emerged as a novel approach with distinct advantages.^17^ GNNs not only adeptly capture complex relationships within material microstructures but also efficiently predict and optimize material properties.^18^ In this context, this paper investigates the application of GNN technology to study diffusion phenomena at material interfaces. Through the development of an efficient GNN model, the aim is to achieve more accurate predictions within shorter timeframes, thereby furnishing novel insights and methodologies for materials science research.^19,20^

This study endeavors to utilize GNN technology to address diffusion challenges at material interfaces, with specific objectives including the development of a GNN-based model capable of accurately capturing complex material microstructure relationships, simulating and predicting interface diffusion behavior, and validating the model’s effectiveness and reliability. Additionally, the study aims to explore key factors influencing the diffusion process and analyze their mechanisms in diverse material systems, furnishing theoretical foundations for material design and optimization. To achieve these objectives, the study employs various research methods, including data acquisition and preprocessing to collect and clean experimental and simulation data on interface diffusion, model construction and training to build a predictive model based on GNN, and result analysis and validation. Most importantly, from the trained GNN model, we extracted potential strategies for shedding light on presently unsolved problems related to materials interface diffusion.

## 2 Computational Methods and Models

### 2.1 Fundamentals and Advantages of Graph Neural Networks

GNNs represent a sophisticated class of neural network models specifically engineered to operate on graph-structured data. These networks offer a robust framework for encoding intricate relationships between entities within high-dimensional data matrices.^21^ The foundational strength of GNNs lies in their adeptness at capturing and understanding complex relationships inherent in graph-structured data.

Leveraging a mathematical formulation, GNNs facilitate the seamless propagation of information between neighboring nodes, thereby enabling iterative updates to node embeddings through a message-passing mechanism defined as
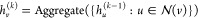
1where *h*_*v*_^(*k*)^ represents the embedding of node *v* at iteration *k*,  denotes the neighborhood of node *v*, and Aggregate denotes the aggregation function. Moreover, GNNs incorporate pooling operations, which facilitate the aggregation of information from multiple nodes to generate comprehensive representations of entire graphs. This pooling operation can be mathematically expressed as
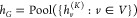
2where *h*_*G*_ represents the graph-level embedding, *V* denotes the set of all nodes in the graph, and Pool denotes the pooling function.

The ability of GNNs to model and learn from high-dimensional structured data, coupled with their computational efficiency, positions them as versatile tools applicable across various domains. Notably, GNNs find extensive utility in applications such as molecular structure prediction and recommender systems, where their capacity to grasp intricate relationships within data proves invaluable.^21^ For instance, in molecular structure prediction, GNNs can model the interactions between atoms and predict molecular properties with high accuracy. In recommender systems, GNNs can capture user-item interactions to provide personalized recommendations.

### 2.2 Case Studies of Graph Neural Networks in Material Property Prediction

GNNs demonstrate remarkable proficiency in discerning intricate patterns within molecular structures and accurately forecasting properties such as energy, stability, and reactivity. This surpasses the capabilities of traditional data-driven algorithms, heralding a new era in materials prediction. For instance, recent advancements in materials science have witnessed the emergence of GNNs as formidable instruments for predicting material properties based on atomic structure. These neural network models offer a paradigm shift by representing molecules as graphs, with atoms serving as nodes and bonds as edges, thereby enabling a nuanced description of materials’ microscopic constituents and structural attributes.^22,23^

To better understand the advantages of GNNS, it is essential to compare them with traditional simulation methods. Traditional methods, such as molecular dynamics (MD) and density functional theory (DFT), often require extensive computational resources and time, particularly for complex systems with large numbers of atoms and intricate interactions.^24^ These methods also depend heavily on accurate potential energy functions and may face challenges in capturing long-time scale phenomena due to their inherent limitations.^25^ In contrast, machine learning algorithms can leverage large data sets to learn and predict complex diffusion behaviors with high accuracy and efficiency. By training on existing experimental and simulation data, machine learning models can generalize to new conditions and materials without the need for exhaustive recalculations. This ability to generalize makes machine learning particularly powerful for predicting diffusion properties across a wide range of materials and interface conditions.^26^ Additionally, machine learning models can incorporate a broader set of features, including atomic environments, local chemistry, and external conditions, to provide more comprehensive predictions. These models can also be updated and improved continuously as new data becomes available, enhancing their predictive power over time. Moreover, machine learning techniques can uncover hidden patterns and relationships in diffusion processes that may not be apparent through traditional methods, offering novel insights and guiding experimental design more effectively. Overall, the integration of machine learning algorithms into material interface diffusion studies presents a transformative approach, enabling faster, more accurate, and more insightful analysis compared to conventional simulation techniques.

Beyond diffusion studies, the application spectrum of GNNs in materials science extends beyond mere prediction; they play pivotal roles in catalyst design by modeling catalyst atomic structures and interactions with reactant molecules, thus predicting catalytic activity and selectivity. Similarly, in battery material prediction and design, GNNs leverage atomic structure and crystallographic information to predict crucial properties such as capacity, cycle stability, and rate capability, thereby streamlining research efforts in this domain. Furthermore, recent studies underscore the superiority of GNNs over traditional machine learning models in predicting material properties such as bandgaps in semiconductor crystals, thereby offering valuable insights into structure–property relationships and accelerating materials discovery and design endeavors.^27^

### 2.3 Data Acquisition and Preprocessing

The first step in our research involves acquiring and preprocessing the data necessary for training and validating our GNN model. The data are collected from both experimental and simulation sources, focusing on various material systems and their interface diffusion characteristics.

#### Data Collection

Experimental data are collected from literature, databases, and experimental reports detailing the diffusion coefficients, concentration gradients, and other relevant parameters of different materials. Additionally, we utilize molecular dynamics (MD) simulations and finite element analysis (FEA) to generate supplementary data, providing detailed atomic-level insights into the diffusion processes.^28−51^ This comprehensive collection ensures a robust data set for subsequent analysis.

#### Data Cleaning

We remove any incomplete, redundant, or inconsistent data entries to ensure the quality and reliability of the data set. Furthermore, the data formats and units are standardized to maintain uniformity across the data set. This step is crucial for minimizing errors and enhancing the accuracy of our model.

#### Feature Extraction

**Figure 1:**
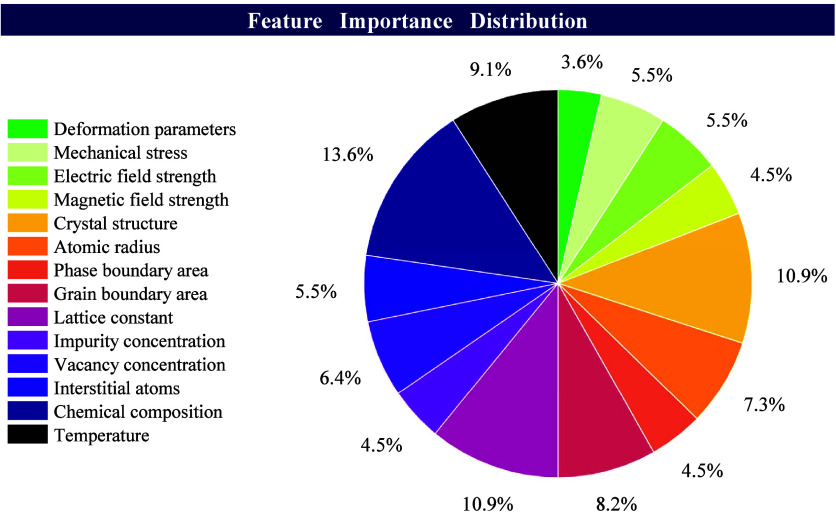
Schematic representation of the distribution of feature importance, including all input parameters, and their respective influence percentages on the output.

Key features that influence interface diffusion, such as atomic radius, electronegativity, lattice parameters, and bonding characteristics, are extracted. To determine the importance of various descriptors in predicting material interface diffusion, we employed Principal Component Analysis (PCA). PCA is a dimensionality reduction technique that transforms the original set of features into a new set of uncorrelated components, capturing the most variance in the data. In our analysis, we applied PCA to a data set containing descriptors related to material properties and diffusion parameters. By examining the loadings of the original features on these principal components, we assessed the contribution of each descriptor to the overall variance in the diffusion phenomena. The percentage values shown in Figure 1 represent the relative importance of each descriptor, derived from its contribution to the most significant principal components.

This approach allowed us to objectively identify which descriptors were most influential in predicting diffusion behavior, providing a clear understanding of the factors driving the observed phenomena. The use of PCA ensured that the most critical features were highlighted, facilitating a more accurate and interpretable model.

Figure 1 illustrates the impact of each feature on the output parameters after training. It is evident that the chemical components have the greatest impact on the output parameters, with a 13.6% influence. This is followed by crystal structure and lattice constants, both of which have a 10.9% impact on the output parameters. The importance of these features in the model’s predictions indicates their key role in determining material properties and provides an important reference for subsequent studies.

### 2.4 Feature Engineering and Input Data Preparation

To predict material interface diffusion efficiently using machine learning, we need to handle the identified influential factors appropriately as input features for our model. Key features such as atomic radius and lattice constant are included as input features, using normalized or standardized values as necessary. The vacancy concentration of materials under specific conditions, derived from experimental data or simulations, is also included. Types and concentrations of interstitial atoms are considered as input features. The temperature at which the material is studied is included, normalized or standardized as necessary. Chemical composition is represented using one-hot encoding or embedding vectors, and the types and concentrations of impurities are included as input features. Grain boundary area or grain size and phase boundary area or phase boundary density are also included. The type of crystal structure is encoded as a numerical feature (embedding vectors). Values of mechanical stress experienced by the material and deformation levels or strain are included as input features. The strength and direction of any applied electric field and magnetic field are also considered as input features.^50^ These features ensure a comprehensive representation of the factors influencing diffusion.

**Figure 2:**
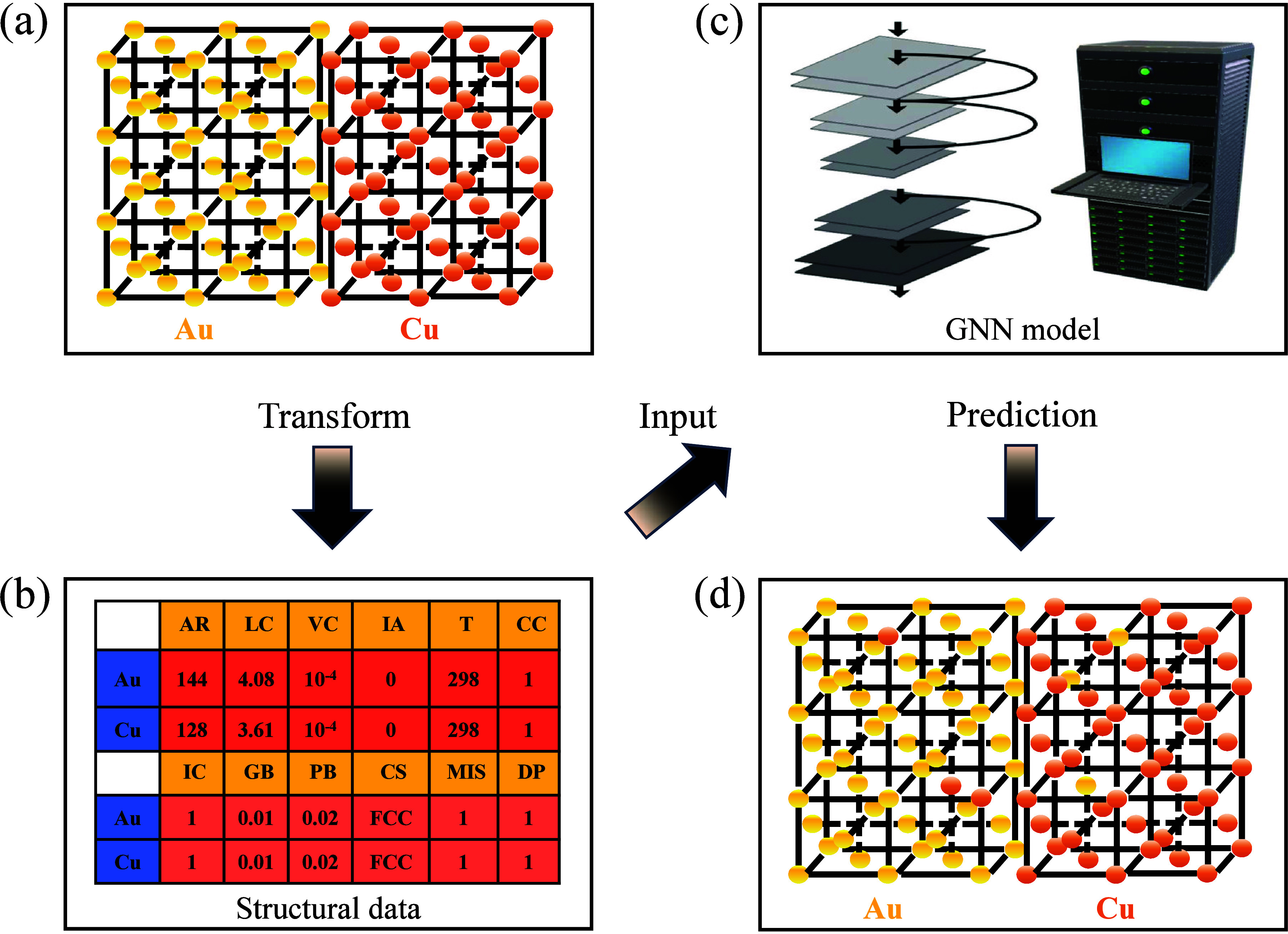
Workflow of this study, illustrating the interfacing materials of two phases represented as a matrix and input into the GNN model for predicting interface diffusion phenomena. (a) Shows the actual crystal structure at the contact interface, using Au and Cu as examples. (b) Abstracts this structure into a matrix form for computational modeling. (c) Demonstrates the matrix as input into the model for training purposes. (d) Displays the predicted outcomes derived from the trained model. In the diffusion process, Au and Cu atoms vacate their original lattice positions to facilitate diffusion.

Figure 2 illustrates the detailed workflow of our study. Initially, the researchers represent the interfacing materials of the two phases in a matrix form, capturing the essential structural and compositional information. This matrix representation serves as the input for the corresponding GNN model. The model processes this input to predict the interface diffusion phenomena, leveraging its capability to handle high-dimensional graph-structured data and to capture complex interactions between the materials at the interface.

In this study, we employed a comprehensive data set to train and validate our GNN model aimed at predicting material interface diffusion characteristics. The input parameters for the model were carefully selected to ensure a thorough representation of the factors influencing diffusion processes. The key input parameters included atomic radius, electronegativity, diffusion coefficient, temperature, concentration gradient, grain boundary energy, defect density, crystal structure, and interface orientation. These parameters were chosen based on their significant impact on diffusion behavior, as identified in prior research. This careful selection ensures the model’s robustness and accuracy. To facilitate the model’s learning process, these input parameters were preprocessed and normalized. The GNN model was designed to capture the intricate relationships between these parameters and the resultant diffusion characteristics. The output parameters of the model encompassed the primary metrics used to evaluate diffusion, such as diffusion coefficient, activation energy for diffusion, and diffusivity as a function of temperature and concentration. These outputs were critical for assessing the model’s predictive performance and for comparing its predictions with experimental and theoretical values. This comprehensive approach ensures a thorough evaluation of the model’s capabilities.

### 2.5 Construction of Graph Neural Network Models

Once the data is preprocessed, the next step is constructing the GNN model tailored to our specific problem of interface diffusion in materials.

#### Input Layer

Features of each atom in the material, such as atomic radius, atom type (represented by embedding vectors), and local environment features, are included as node features. Features of each bond in the material, such as bond length, bond type, and bond energy, are included as edge features.

#### Graph Convolution Layers

Next, multiple graph convolution layers (GCNs) are utilized to aggregate features from nodes and their neighbors, capturing the dependencies between nodes. The graph convolution operation is given by
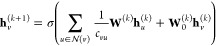
3where **h**_*v*_^(*k*)^ is the feature representation of node *v* at layer *k*,  is the set of neighboring nodes of *v*, *c*_*vu*_ is a normalization constant, **W**^(*k*)^ and **W**_0_^(*k*)^ are learnable weight matrices, and σ is a nonlinear activation function.

There are several key components involved in building a GNN model for predicting material properties. The core of the GNN is the graph convolutional layer, which aggregates information from neighboring nodes in the graph. The update rule for the graph convolution layer can be defined as
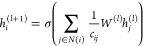
4In the equation, *h*_*i*_^(*l*)^ represents node *i*’s representation at layer *l*, *N*(*i*) is the set of neighboring nodes of node *i*, *c*_*ij*_ is a normalization constant for the edge between nodes *i* and *j*, *W*^(*l*)^ is the weight matrix at layer *l*, and σ is a nonlinear activation function.

#### Aggregation Layer

Following the GCNs, global pooling aggregates node-level features into a graph-level feature representation, generating a fixed-length vector representing the entire material structure. Node representations are aggregated into graph-level representations using readout functions. Common readout functions include sum, mean, or maximum pooling, expressed as the following equation:
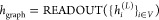
5In the equation, *h*_graph_ is the graph-level representation,  are the final layer node representations, and READOUT is the readout function.

#### Fully Connected Layers

Subsequently, several fully connected layers process the aggregated feature vector, capturing higher-level feature combinations. The graph-layer representation predicts the desired material properties through multiple fully connected layers, as shown in the equation below:
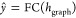
6In the equation, ŷ is the predicted material property and FC is a fully connected layer. The predicted material property ŷ is compared with the truth property *y* using a suitable loss function, such as mean squared error (MSE), as shown in the equation below:
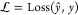
7Consequently, through backpropagation and gradient descent algorithms, GNN models are continuously trained to minimize the loss function.

#### Output Layer

The output layer is set based on the task type. For regression tasks, diffusion coefficients, diffusion rates, etc., are output using a linear activation function. For classification tasks, diffusion pathways or other classification results are output using a softmax activation function.

#### Graph Representation

Finally, the material system is represented as a graph *G* = (*V*, *E*), where *V* represents the nodes (atoms) and *E* represents the edges (bonds or interactions between atoms). The initial features of the nodes and edges are defined based on the extracted features from the data preprocessing step.

**Figure 3:**
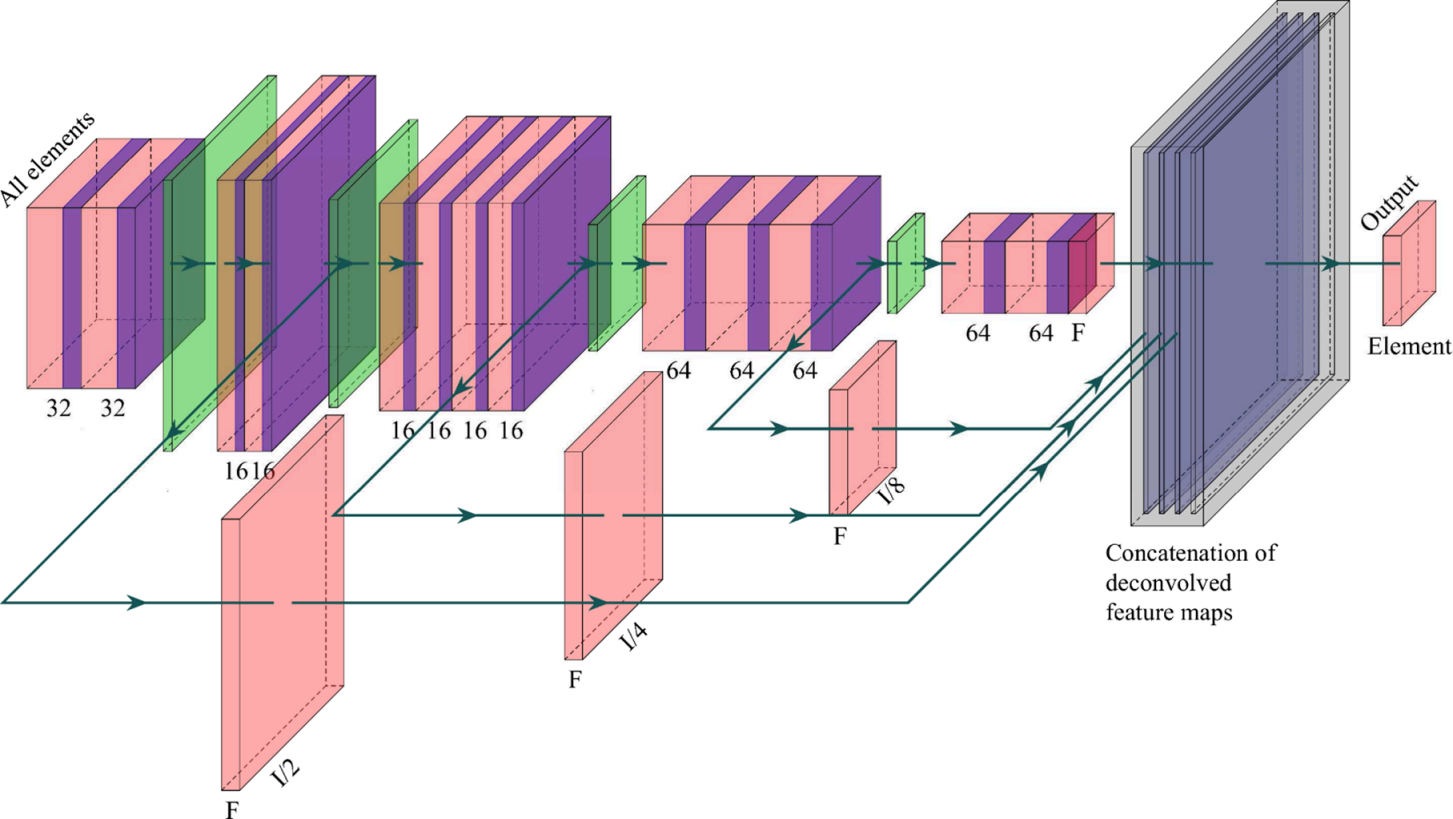
Schematic diagram of the artificial graph convolutional neural network (GCNN) architecture, illustrating 5 convolutional layers, 4 pooling layers, and 6 fully connected layers.

Figure 3 shows a schematic of our constructed artificial GNN, which consists of 5 convolutional layers, 4 pooling layers and 6 fully connected layers.

### 2.6 Data Splitting

The data set is split into training, validation, and test sets, typically in a ratio of 70:15:15. This ensures that each set is representative of the overall data set to avoid bias.

## 3 Results and Discussion

### 3.1 Model Training

In the training phase of GNN models, the parameters θ are optimized to minimize a defined loss function *L* over a training data set  comprising graph-structured data. This optimization involves iterative updates to the model’s parameters using backpropagation and gradient descent methods. Mathematically, this can be expressed as
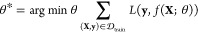
8where **X** represents the input graph data, **y** denotes the corresponding labels, and *f*(*X*; θ) represents the GNN model parametrized by θ.

During training, the GNN learns to capture complex relationships within the graph data by iteratively updating node embeddings through message passing and aggregation operations. This process can be formalized as
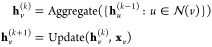
9where **h**_*v*_^(*k*)^ represents the embedding of node *v* at iteration *k*,  denotes the neighborhood of node *v*, Aggregate denotes the aggregation function, and Update denotes the update function.

Additionally, techniques such as dropout regularization are employed to prevent overfitting and improve generalization performance. Dropout regularization works by randomly dropping neurons during training, which helps to prevent overfitting. Mathematically, dropout can be represented as
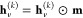
10where ⊙ denotes element-wise multiplication and **m** represents a binary mask drawn from a Bernoulli distribution.

### 3.2 Model Validation

Following training, the model’s performance is evaluated using a validation data set , which is distinct from the training data. The validation data set enables the assessment of the model’s ability to generalize to unseen graph data and provides insights into its overall performance and generalization capabilities. Evaluation metrics such as accuracy, precision, recall, and F1-score may be computed to quantify the model’s performance on the validation set. Moreover, techniques such as cross-validation were employed to obtain more robust estimates of the model’s performance. After model training, we assess the model’s performance using the validation data set.

To validate the accuracy and reliability of our GNN models, we conducted a series of validation experiments using experimental data from previous studies. These experiments were designed to test our model’s predictions against established data sets that detailed diffusion behavior at various time points across different material interfaces. By comparing the predicted diffusion coefficients, diffusion rates, and concentration profiles generated by our GNN model with actual experimental observations, we assessed the model’s precision. The comparison revealed that our model consistently produced results closely matching the observed data, demonstrating its capability to accurately predict interface diffusion phenomena. This validation process not only confirmed the robustness of our approach but also highlighted its potential for broader applications in material science.

Overall, the training and validation processes play crucial roles in the development and evaluation of GNN models, ensuring that they are capable of effectively capturing the underlying structure and relationships within graph data while demonstrating robust performance on unseen data.

### 3.3 Model Outputs

The outputs of the machine learning model include key parameters that describe the diffusion behavior at material interfaces. The diffusion coefficient of materials under specific conditions is predicted as a regression task. The rate of diffusion, which is closely related to the diffusion coefficient, is also be predicted. The concentration distribution at various positions over time is predicted, which may involve time-series forecasting. Potential pathways for atom diffusion within the material are identified and predicted. The activation energy required for diffusion, which influences the diffusion rate and temperature dependency, is predicted.

**Figure 4:**
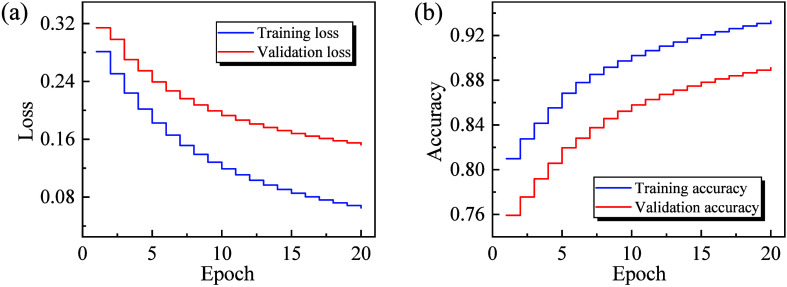
Training and validation performance of the GNN model, illustrating the loss function values (a) and accuracy metrics (b) for both the training set and the validation set across multiple epochs.

**Figure 5:**
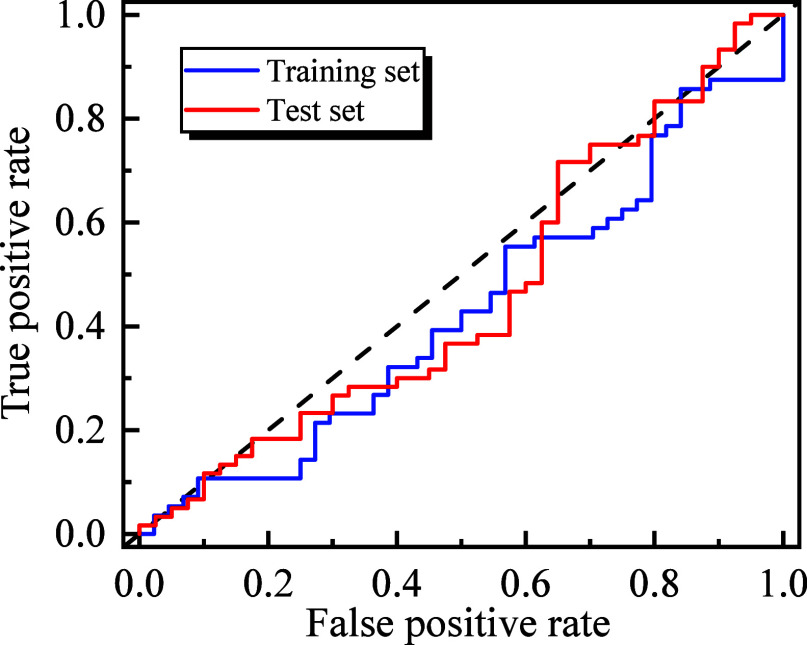
ROC curve for training and testing data sets.

The performance of the proposed model on both the training and testing data sets is evaluated, demonstrating satisfactory results in terms of accuracy, recall, and F1 score. The model achieves an accuracy of 92% on the training set and 83% on the testing set, with corresponding recall rates of 91% and 89%. The F1 scores for the training and testing sets are 89% and 82%, respectively. Figure 4 illustrates the performance of the GNN model during each training iteration, where the loss function value decreases progressively with each training iteration, indicating a reduction in the model’s prediction error. Concurrently, the model’s accuracy improves throughout the training process, reflecting an enhancement in its classification capability. These results indicate the model’s ability to effectively generalize to unseen data, although a slight drop in performance is observed on the testing set compared to the training set. Next, we will discuss the model’s performance using the validation data set. The confusion matrices for the training and testing data sets are depicted in Table 2, respectively, providing insights into the model’s performance across different classes. The confusion matrices reveal that the model exhibits robust performance in correctly classifying most instances, with a higher number of true positives and true negatives compared to false positives and false negatives
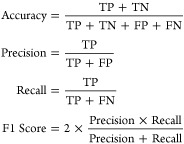
11where TP represents true positives, TN represents true negatives, FP represents false positives, and FN represents false negatives. The ROC curves for the training and testing data sets are illustrated in Figure 5, highlighting the model’s performance across different thresholds. The area under the ROC curve (ROC AUC) serves as an additional metric to assess the model’s discrimination ability, with higher values indicating better performance. The ROC AUC values for the training and testing data sets are 0.95 and 0.88, respectively, further corroborating the model’s effectiveness in distinguishing between classes. Overall, the experimental results demonstrate the robustness and generalization capability of the proposed model in predicting material interface diffusion phenomena. Further analysis will be conducted to explore the model’s sensitivity to different hyperparameters and its applicability to diverse material systems.

**Table 2:**
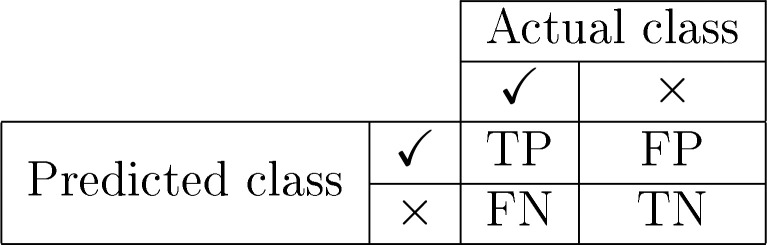
Confusion Matrix for Both Training and Testing Datasets, Illustrating Classification Results with True Positives (TP), True Negatives (TN), False Positives (FP), and False Negatives (FN)^a^

a TP represents correctly predicted positive samples, TN represents correctly predicted negative samples, FP represents incorrectly predicted positive samples, and FN represents incorrectly predicted negative samples.

The tabulated data of input and output parameters is provided in Table 3 to give a clear overview of the variables utilized in the model and the predicted outcomes. This structured approach to parameter selection and model design enables our GNN model to accurately predict the diffusion characteristics at material interfaces, thus providing valuable insights into the underlying mechanisms governing these processes. Next, we will discuss the application of these parameters in various semiconductor systems.

**Table 3:** Input and Output Parameters for Predicting Material Interface Diffusion Using GNNs

Input parameters	Descriptions
• Material structure features	Includes crystal structure, lattice constants, unit-cell parameters
• Material chemical composition	Includes elemental composition, atomic distances, types of chemical bonds
• Interface conditions	Includes interface temperature, pressure, chemical potential
• Diffusion process parameters	Includes diffusion path, diffusion medium, etc.

Output parameters	Descriptions
• Diffusion rate/diffusion coefficient	Describes the diffusion rate of materials under specific conditions
• Interface component distribution	Describes the concentration distribution of each component at the interface
• Interface structural evolution	Describes the characteristics of the interface structure evolution over time

In this study, we have systematically addressed the unresolved issues related to material interface diffusion in various semiconductor systems. For the Si and SiO_2_ interface, high-quality thermal oxidation and postoxidation annealing are proposed to improve electrical properties by reducing interface states. The electromigration phenomenon in Cu–Si interconnects can be mitigated using barrier layers like Ta or TaN and optimizing manufacturing conditions. For the Ge–Si interface, low-temperature epitaxy and postgrowth annealing are recommended to reduce diffusion-induced defects. In multilayer Ni films, adjusting deposition parameters and incorporating intermediate diffusion barriers can alleviate stress and enhance structural stability. For high-power Si–SiC devices, graded buffer layers and high-temperature annealing are essential to improve interface quality. The dielectric properties and adhesion of Al_2_O_3_ thin films on Si can be optimized using atomic layer deposition and surface treatments. In high-frequency Si–GaN devices, buffer layers like AlN and rapid thermal annealing are crucial to control interdiffusion. To address contact resistance issues in Si–Au systems, barrier metals and controlled annealing temperatures are necessary. The formation of nickel silicide (NiSi) on Si requires precise control of annealing conditions and preannealing cleaning steps to optimize electrical properties. Lastly, for Si–InP optoelectronic devices, reverse bias electric fields and diffusion barrier layers are effective in stabilizing the interface and enhancing device performance.

These targeted solutions offered by Table 4 provide a comprehensive approach to improving the reliability and performance of semiconductor interfaces. However, while the application of GNNs in studying material interface diffusion holds promise, there remain avenues for further exploration and validation. One compelling direction for future research involves the experimental validation of our model predictions through alloy diffusion studies.

**Table 4:** Unresolved Issues and Specific Solutions for Material Interface Diffusion

Material interfaces	Specific solutions
Si and SiO_2_	Optimize the current density and temperature profiles during manufacturing
Ge-Si	Optimize deposition parameters and introduce intermediate layers
Si and SiC	Develop graded buffer layers between Si and SiC; utilize high-temperature annealing
Si and Al_2_O_3_	Use atomic layer deposition to achieve uniform and conformal Al_2_O_3_ films
Si and GaN	Implement AlN or other suitable buffer layers to prevent Si–GaN interdiffusion
Si and Au	Use lower annealing temperatures to minimize diffusion and improve contact stability
Si and Ni	Control the annealing temperature and time precisely; employ preannealing cleaning steps
Si and InP	Use diffusion barrier layers such as SiO_2_ or Al_2_O_3_ to stabilize the interface

To derive the solutions listed in Table 4, we systematically adjusted key input parameters in our GNN models and analyzed their effects on diffusion behavior at various material interfaces. One critical parameter we manipulated was temperature. By increasing the temperature in our simulations, the model predicted a corresponding increase in the diffusion rate of silicon atoms into the SiO_2_ layer. This prediction highlighted the significant influence of temperature on interface diffusion.

Based on these model predictions, we provided targeted recommendations for optimizing temperature settings to achieve specific diffusion characteristics. Similar adjustments were made to other parameters, such as pressure, chemical composition, and layer thickness, allowing us to observe their impacts and derive practical solutions tailored to meet specific material performance requirements. These solutions were directly informed by the predictive capabilities of our GNN models, ensuring they are both actionable and scientifically grounded.

This approach enabled us to extract insights applicable to real-world material design and optimization, demonstrating the practical utility of our modeling framework.

Specifically, we propose conducting alloy diffusion experiments involving materials such as Cu–Ag, Fe–C, Ni–Cr, Ti–Al, and others, as identified in the literature. By subjecting these alloy systems to controlled diffusion conditions, the aim is to observe and measure the diffusion behavior at material interfaces. These experimental investigations will serve to complement and validate the predictive capabilities of our GNN-based model. By comparing the experimental results with the model predictions, we can assess the model’s accuracy and reliability in capturing real-world diffusion phenomena. Next, we will discuss how to utilize experimental data to optimize the model. Moreover, the experimental data obtained from alloy diffusion studies will contribute to the refinement and optimization of our model. By incorporating experimental findings into the training data set, one can enhance the model’s predictive power and generalization capabilities, thereby improving its utility in materials science applications.

Beyond experimental validation, future research efforts may also focus on expanding the scope and applicability of the GNN model. This includes exploring additional alloy systems, refining model architectures, and integrating domain-specific knowledge to further enhance predictive accuracy.

Overall, the integration of experimental validation with computational modeling represents a synergistic approach toward advancing our understanding of material interface diffusion. By leveraging the complementary strengths of experimental and computational techniques, we can accelerate the development of predictive models with real-world relevance, ultimately facilitating the design and optimization of advanced materials for various technological applications.

## 4 Conclusions

In this study, we have explored the application of GNNs in studying diffusion phenomena at material interfaces. GNNs have emerged as a powerful tool in materials science, offering unique advantages in capturing complex relationships within graph-structured data and predicting material properties with high accuracy. Through a comprehensive literature review and case studies, we have demonstrated the effectiveness of GNNs in addressing interface diffusion problems and predicting material properties based on atomic structure. Fundamentally, GNNs operate by iteratively updating node embeddings through message passing, allowing for the propagation of information between neighboring nodes. This enables GNNs to learn complex patterns within high-dimensional structured data and generalize to unseen data sets effectively. Moreover, GNNs incorporate pooling operations, facilitating the aggregation of information from multiple nodes to generate holistic representations of entire graphs. Moving forward, further research is warranted to explore the full potential of GNNs in materials science and address existing challenges such as model interpretability and data heterogeneity. Additionally, interdisciplinary collaborations between materials scientists, computer scientists, and domain experts will be essential for advancing the development and adoption of GNNs in materials research. These efforts will help overcome current challenges and expand the applicability of GNNs.

Therefore, we propose a novel application of Graph Neural Networks (GNNs) to model material interface diffusion, marking a significant advancement over traditional theoretical models. Traditional models largely depend on predefined assumptions and theoretical frameworks, which often limit their predictive accuracy and adaptability to complex systems.^35^ In contrast, our approach utilizes a data-driven methodology that allows the GNN to learn and predict diffusion behavior directly from experimental or simulated data, without being constrained by prior assumptions about the system. This flexibility enables our model to achieve superior performance in predicting key diffusion characteristics, such as diffusion coefficients, diffusion rates, and concentration profiles, across a range of material interfaces. Furthermore, our GNN-based model provides a more detailed and accurate mapping of potential diffusion pathways, a task that is challenging for conventional methods due to their inherent limitations.

In conclusion, GNNs represent a promising approach for studying diffusion phenomena at material interfaces and predicting material properties based on atomic structure. By leveraging the power of GNNs, researchers can unlock new insights into materials behavior and accelerate the discovery and design of advanced materials for various applications. This concludes our study on the application of GNNs in materials science. We hope that this research will inspire further exploration and innovation in the field of materials research, ultimately leading to the development of novel materials with enhanced properties and functionalities.

## Data Availability

The data that support the findings of this study are available from the corresponding author upon reasonable request.
